# Virtual screening and molecular dynamics simulations provide insight into repurposing drugs against SARS-CoV-2 variants Spike protein/ACE2 interface

**DOI:** 10.1038/s41598-023-28716-8

**Published:** 2023-01-27

**Authors:** Davide Pirolli, Benedetta Righino, Chiara Camponeschi, Francesco Ria, Gabriele Di Sante, Maria Cristina De Rosa

**Affiliations:** 1 Institute of Chemical Sciences and Technologies ‘‘Giulio Natta’’ (SCITEC)-CNR, 00168 Rome, Italy; 2 Department of Translational Medicine and Surgery, Section of General Pathology, Università Cattolica del Sacro Cuore, 00168 Rome, Italy; 3 Fondazione Policlinico Universitario A. Gemelli IRCCS, 00168 Rome, Italy; 4 Department of Medicine and Surgery, Section of Human, Clinic and Forensic Anatomy, University of Perugia, 06132 Perugia, Italy

**Keywords:** Cheminformatics, Virtual screening

## Abstract

After over two years of living with Covid-19 and hundreds of million cases worldwide there is still an unmet need to find proper treatments for the novel coronavirus, due also to the rapid mutation of its genome. In this context, a drug repositioning study has been performed, using in silico tools targeting Delta Spike protein/ACE2 interface. To this aim, it has been virtually screened a library composed by 4388 approved drugs through a deep learning-based QSAR model to identify protein–protein interactions modulators for molecular docking against Spike receptor binding domain (RBD). Binding energies of predicted complexes were calculated by Molecular Mechanics/Generalized Born Surface Area from docking and molecular dynamics simulations. Four out of the top twenty ranking compounds showed stable binding modes on Delta Spike RBD and were evaluated also for their effectiveness against Omicron. Among them an antihistaminic drug, fexofenadine, revealed very low binding energy, stable complex, and interesting interactions with Delta Spike RBD. Several antihistaminic drugs were found to exhibit direct antiviral activity against SARS-CoV-2 in vitro, and their mechanisms of action is still debated. This study not only highlights the potential of our computational methodology for a rapid screening of variant-specific drugs, but also represents a further tool for investigating properties and mechanisms of selected drugs.

## Introduction

More than 628 million cases (COVID-19) caused by the infection with Coronavirus 2019 (SARS-CoV-2) have been reported so far according to the World Health Organization (https://covid19.who.int/ last website visit, November 4th 2022), with an increased and probably underestimated number of cases associated to the spread of its variants. Specifically, the mutations of the receptor binding domain (RBD) of the Spike (S) glycoprotein, have caused great concern in this pandemic^1–3^ and their prediction, prevention and treatment still remain the main unmet needs that science is globally challenged to address. The S protein of SARS-CoV-2 plays a key role in viral infection, with the S1 domain responsible for receptor binding and the S2 domain mediating membrane fusion^4^. Similarly to SARS-CoV and other related viruses, S glycoprotein engages the angiotensin-converting enzyme 2 (ACE2) to penetrate into the host cell^5^. This ability is stronger in SARS-CoV-2 than in SARS-CoV due to mutations of the RBD within S protein, which could account for the increased infectivity and transmissibility^6^.

Since the pandemic has progressed, spread, and circulated worldwide, several different variants of concern (VoCs) have emerged, namely, the Alpha (B.1.1.7), Beta (B.1.351), Gamma (P.1), Delta (B.1.617.2) and Omicron (B.1.1.529). All these VoCs, excepting the Delta, share and harbor the same N501Y mutation of the S protein, which results in an increased affinity of Spike protein for ACE2 receptors^7^. The Delta variant carries three other key S mutations: (i) L452R, which may stabilize the interaction between S protein and ACE2, consequently enhancing infectivity^8^; (ii) T478K, which has been correlated with an increment in the positivity rate^9^; (iii) P681R, which has been shown to facilitate the fusion activity and therefore may promote transmissibility^10^. On the whole the SARS-CoV-2 Delta VoC is 40–60% more transmissible than the Alpha (B.1.1.7) VoC and has been associated with an increased risk of hospitalization^11^. The same recently discovered Omicron variant, which in a short time has become the dominant strain of COVID-19 globally, exhibits a significantly lower risk of serious disease^12^.

The Omicron variant(s) has(have) a significantly larger number of mutations in the RBD compared to Delta variant, ten of which (N440K, G446S, S477N, T478K, E484A, Q493R, G496S, Q498R, N501Y, and Y505H) occur in the main functional motif at the interface between S protein and ACE2 known as receptor-binding motif (RBM)^13^. The substantial variation in the number and pattern of mutations has been associated to the increased transmissibility of the Omicron variant(s)^14^.

To date the FDA did not approve any specific drugs against COVID-19 with the exception of Remdesvir and few medications for emergency use, including some antiviral drugs (paxlovid), kinase inhibitors (e.g., baricitinib), and monoclonal antibodies (casirivimab, imdevimab, sotrovimab, bamlanivimab and etesevimab) for hospitalized adults and children**.** Because of the lack of an effective therapy and limits of vaccine use and efficacy in specific populations, such as children and immunocompromised patients, respectively, there is an unmet need to identify novel therapeutic treatments to counteract and/or prevent SARS-CoV-2 spread.

Among the various strategies, the inhibition of the binding between S protein and ACE2 is a feasible approach to discover or repurpose promising candidate therapies. Various virtual screening methods of FDA-approved drugs, natural and synthetic compounds as potential S protein-ACE2 small-molecule blockers are described in the literature^15–18^. A recent report also illustrated the application of deep learning techniques in structure-based design of inhibitors able to disrupt the binding between SARS-CoV-2-RBD and ACE2^19^. To this end, we previously developed a deep learning-based QSAR model to identify protein–protein interactions (PPI) modulators, to be used for targeting the SARS-CoV-2 Spike RBD/ACE2 interface^20^. Here, our pre-trained QSAR model screened the library of approved drugs to generate a focused library of PPI modulators with existing pharmacokinetic, toxicology and safety data. Virtual screening against Delta variant RBD, followed by molecular dynamics (MD) simulation and MM-GBSA binding energy calculations, to better estimate the binding affinity, has led to the identification of several lead candidates that possess potential therapeutic activity against Delta and may be effective also against the Omicron variant.

The prominence of this work lies not only in the opportunity to identify appropriate drugs quickly and effectively, but also in describing an approach that can reposition drugs already used and approved for other diseases and that can work transversely across multiple variants of SARS-CoV-2.

## Results

### Exploring the druggable binding sites on Delta and Omicron Spike at ACE2-RBD interface

**Figure 1:**
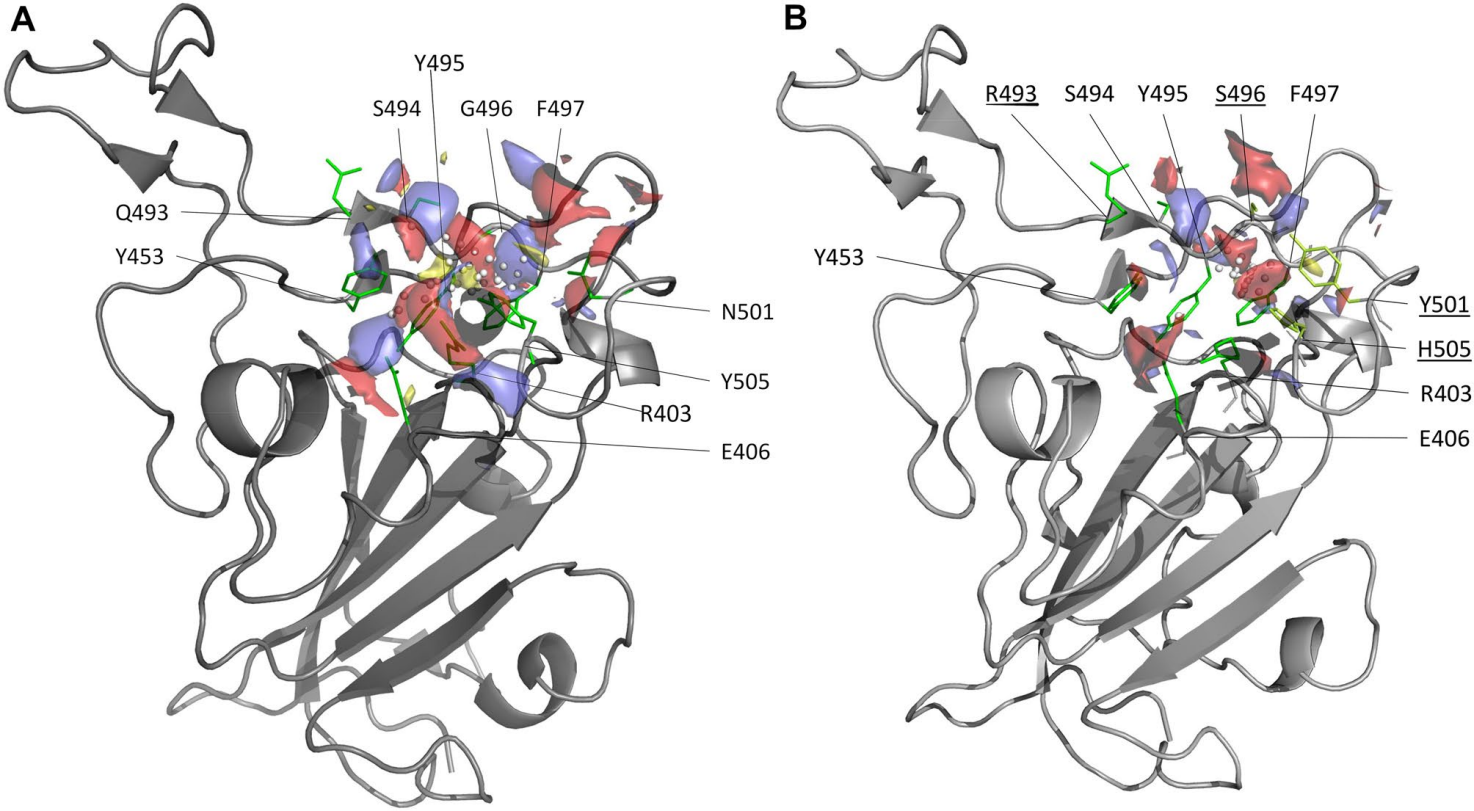
Predicted SiteMap pockets on the Spike surface at the RBD/ACE2 interface. Delta (**A**) and Omicron (**B**) variants are represented as solid grey ribbons and residues lining the pockets are colored in green. Pockets are shown with their relative surfaces, indicating the regions for hydrophobic (yellow), hydrogen bond donor (blue) and acceptor (red) functional groups. Mutated residues in the Omicron variant are underlined.

Our in silico druggability analysis using SiteMap identified in both Delta and Omicron S protein a pocket at the ACE2-RBD interface embedding residues important for the stabilization of the complex (hotspot). Table 1 reports the list of the hotspot residues which were identified through computational alanine scanning mutagenesis, i.e., substituting each residue at the interface with alanine and calculating the corresponding binding free energy change relative to the wild type (∆∆G_bind_). Mutation of hotspot residues to alanine changes the binding free energy of at least 1.0 kcal/mol. In agreement with what has been previously observed for several other PPIs^21,22^, identified ACE2-RBD site had lower score than typical binding pockets (0.6 and 0.5 in Delta and Omicron, respectively). The site was lined by Arg403, Glu406, Tyr495, Gly496 (Ser in Omicron), Phe497, Asn501 (Tyr in Omicron) and Tyr505 (His in Omicron) (Fig. 1) where Asn501 (Tyr in Omicron) and Tyr505 were main hotspots (Table 1). Edge of the larger Delta pocket also included Tyr453, Gln493 and Ser494. The predicted site overlapped with that of Spike RBD identified using FTMap and DeepSite and used as target for docking^23^. Analysis of the non-bonded interactions showed that in both Spike proteins residues at 496, 501 and 505 positions interact with Lys353 and Tyr41 of ACE2. In addition, Delta Gln493 and Omicron Tyr453 of Spike contact ACE2 Glu35 and His34, respectively.

**Table 1:** Hotspot residues.

	Residue number	ΔΔG_binding_ (kcal/mol)
Delta	Y449	1.30
	L455	1.07
	F456	1.77
	F486	1.79
	N487	1.07
	Y489	1.66
	G498	1.63
	N501	1.83
	Y505	3.08
Omicron	Y453	1.63
	F456	1.61
	F486	1.24
	N487	1.16
	Y489	2.09
	R493	1.38
	R498	1.49
	Y501	1.69

Residues with ΔΔG > 1 kcal/mol as calculated by computational alanine scanning.

**Figure 2:**
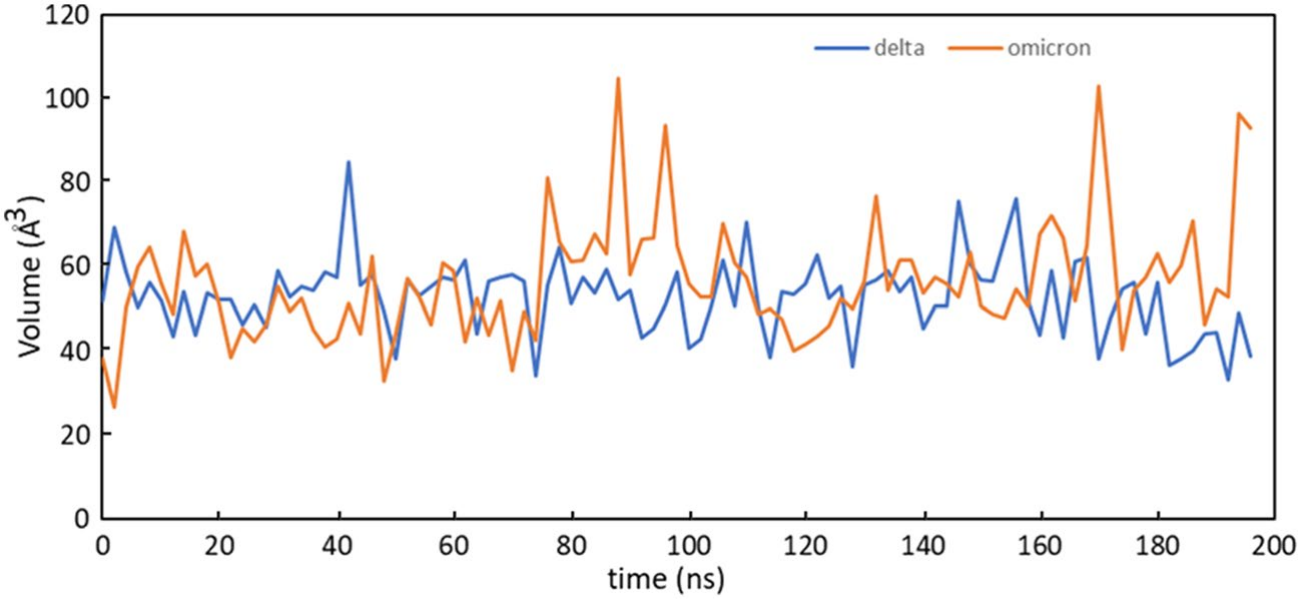
Binding-pocket volumes of Delta (blue) and Omicron (orange) plotted as a function of time.

Given the challenges of identifying druggable sites on PPIs, pockets detected by SiteMap which rely on static protein conformations were assessed by molecular dynamics simulations (MD). The binding site volume was calculated along the MD trajectory and plotted over the simulation time using the script *trajectory_binding_site_volumes.py* available in the Schrödinger suite of software. As evident in the Fig. 2, no significant differences existed in the binding-pocket volumes of the two simulated systems. The pockets were stable over the simulation time and an average binding-pocket volume of 53.0 Å^3^ and 56.5 Å^3^ was calculated for Delta and Omicron variants, respectively. A major fluctuation was observed in Omicron suggesting that its pocket structure may acquire a more expanded conformation as compared to Delta.

### Virtual screening of approved drugs on Delta Spike RBD

**Figure 3:**
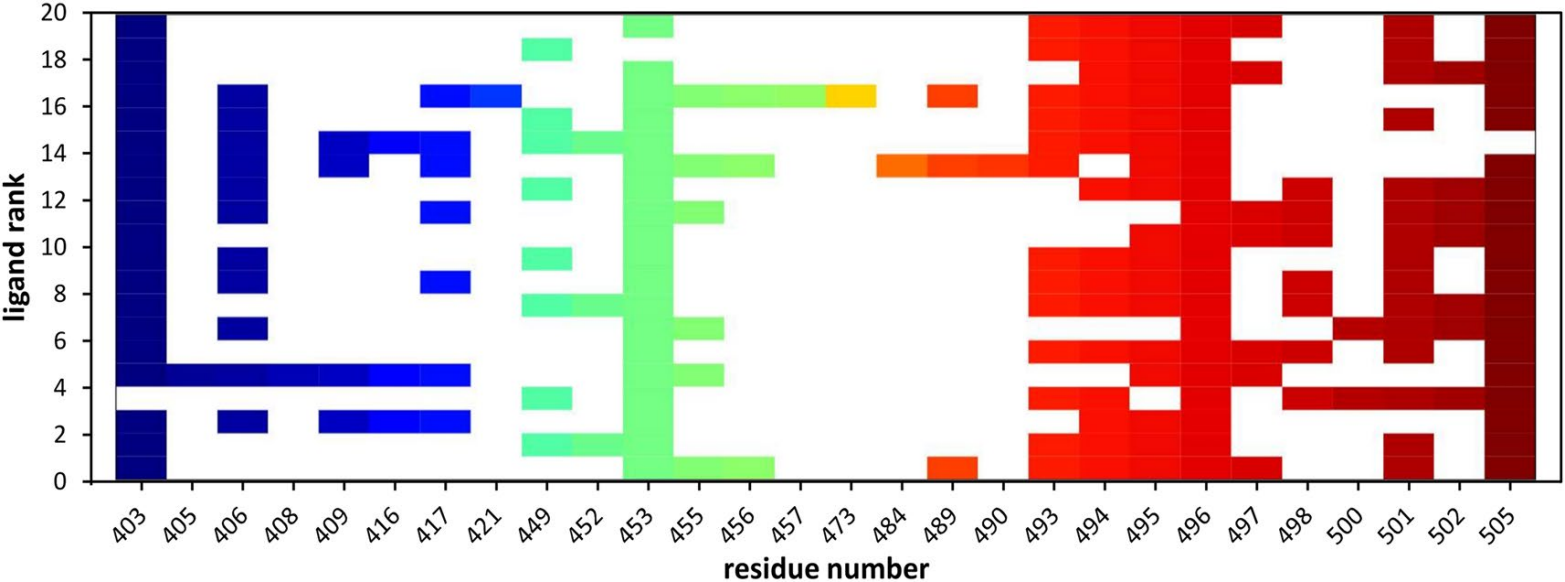
The interaction fingerprint matrix of docking poses into the Delta RBD site. Colored bars indicate one or more interactions between Delta residues (columns) and individual ligands (rows). Different colors indicate different residues.

When we have applied the developed QSAR solution^20^ to screen the library of 4388 approved drugs, we were able to identify 505 potential PPIs modulators. Structure-based virtual screening, with different levels of increasing docking precision, was used to rank the 505 compounds based on their binding affinity. To obtain further accuracy for our protocol^24^, poses of Delta RBD-ligand complexes obtained in Glide XP docking were reranked with MM-GBSA which demonstrated to better distinguish between real and decoy poses of a ligand and assess the energetically preferred pose^25^. The ΔG_bind_ values lower than − 47 kcal/mol were considered to retrieve the final set of molecules, leading to the identification of 20 top-ranked hits. Table 2 reports the calculated ΔG_bind_ values of the selected compounds and the various energy components. Inspection of the free energy components in this Table revealed that for all the compounds the van der Waals and the lipophilic energies (ΔG_vdW_ and ΔG_Lipo_) contribute most to the ligands binding energy. As shown in Fig. 3, an interaction fingerprint (IFP) matrix was generated by Maestro to analyze the binding site residues occurring in any type of contact with the selected 20 compounds. Considering that a cutoff of 1 was used for the number of residue interactions, we found that these 20 ligands made interactions mostly with Arg403, Tyr453, Gly496 and Tyr 505, the last being the main hotspot residue (Table 1).

**Table 2:** Binding energy (kcal/mol) and individual energy terms of Delta RBD-ligand systems calculated by Prime MM-GBSA.

	ligand	ΔG_bind_	ΔG_Coulomb_	ΔG_Covalent_	ΔG_Hbondd_	ΔG_Lipo_	ΔG_SolvGB_	ΔG_vdW_
#1	ZINC000000004319	− 59.54	0.12	1.23	− 2.94	− 13.79	− 15.71	− 26.22
#2	ZINC000003824921	− 54.8	− 37.26	4.57	− 3.47	− 18.48	35.68	− 31.99
#3	ZINC000067665085	− 54.76	− 18.6	1.33	− 2.31	− 17.91	28.05	− 42.13
#4	ZINC000035644633	− 51.87	− 57.74	3.44	− 2.32	− 17.69	61.48	− 34.59
#5	ZINC000085540202	− 51.64	6.63	5.41	− 1.82	− 15.39	3.93	− 47.05
#6	ZINC000000607910	− 51.46	− 21.8	− 0.94	− 2.45	− 13.77	19.15	− 25.85
#7	ZINC000000538509	− 50.78	4.91	2.05	− 1.6	− 17.78	− 3.98	− 30.29
#8	ZINC000053073961	− 50.18	41.13	3.07	− 1.16	− 17.26	− 33.41	− 36.14
#9	ZINC000003810860	− 50.05	− 26.04	4.45	− 1.71	− 19.57	27.55	− 29.34
#10	ZINC000022446634	− 49.42	28.75	5.74	− 1.13	− 17.62	− 29.46	− 32.19
#11	ZINC000003816292	− 48.55	42.49	9.34	− 2.06	− 9.41	− 50.72	− 35.51
#12	ZINC000001530886	− 48.14	31.35	2.7	− 2.59	− 17.89	− 23.82	− 28.71
#13	ZINC000006716957	− 47.99	− 13.17	9.96	− 1.89	− 18.8	21.94	− 38.49
#14	ZINC000077313075	− 47.94	− 63.1	8.9	− 2.92	− 19.73	72.88	− 39.91
#15	ZINC000030731084	− 47.58	− 23.59	5.78	− 4.24	− 13.51	31.38	− 41.64
#16	ZINC000000537928	− 47.34	22.02	3.74	− 0.54	− 26.3	− 11.93	− 29.88
#17	ZINC000150339331	− 47.3	11.6	7.07	− 1.84	− 14.58	− 9.06	− 33.73
#18	ZINC000000001003	− 47.24	− 15.85	2.57	− 1.03	− 15.88	13.98	− 25.87
#19	ZINC000000608266	− 47.12	61.92	5.59	− 0.65	− 22.26	− 55.54	− 32.12
#20	ZINC000001552042	− 47.11	− 77.88	2.11	− 1.33	− 16.25	86.71	− 34.63

### Crystal structure of Delta RBD-ACE2 complex was stable in the MD simulation

**Figure 4:**
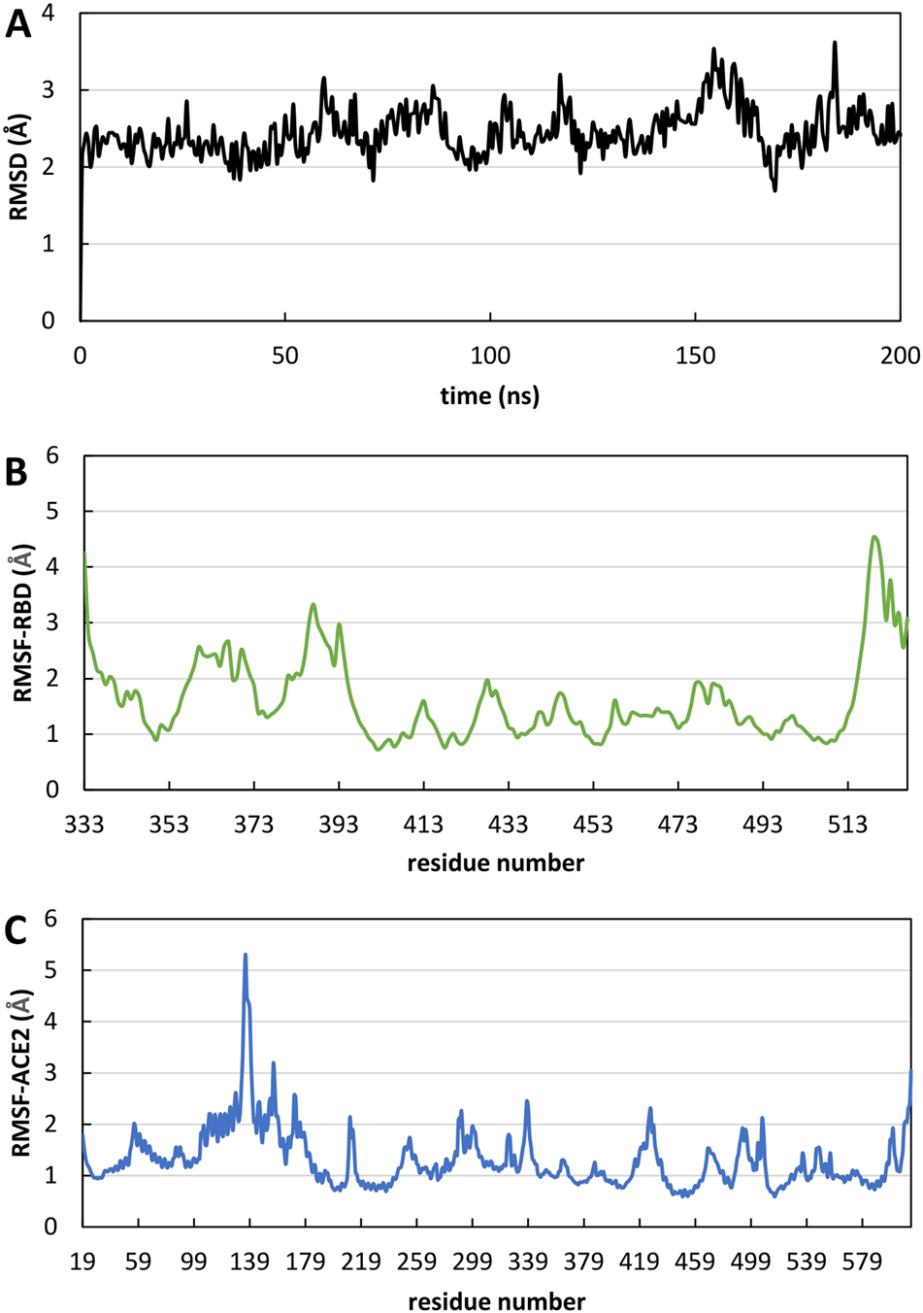
Evolution of structural properties over time. (**A**) Cα-RMSD as a function of time of Delta RBD-ACE2 complex. (**B**) RMSF of Delta RBD as a function of amino acids. (**C**) RMSF of ACE2 as a function of amino acids.

The stability of the RBD-ACE2 complex was analyzed through the root mean square deviation (RMSD) of the simulated protein structure's atomic positions from its native coordinates. The RMSD profile (showed in Fig. 4A) of the Cα atoms remained stable around average values of 2.4 ± 1.7 Å along the entire trajectory indicating that the complex was stable throughout the simulation. Root mean square fluctuation (RMSF) helped to understand the flexibility of each amino acid residue during the simulation time. As already described^26^, the binding interface of Delta RBD-ACE2 complex is made up of two patches of residues. Patch 1 consists of Ser19, Gln24, Phe28, Asp30, Lys31, His34, Leu79, Met82, Tyr83 on ACE2 and of Ala475, Asn487, Gln493, Tyr453, Phe486, Tyr489, Gln498 on RBD. Patch 2 comprises Glu37, Asp38, Tyr41, Gln42, Lys353 on ACE2 and Tyr449, Gly496, Thr500, Tyr505 on RBD. The RMSF analysis showed that slight fluctuations occurred in patches 1 and 2 with RMSF values less than 1.5 Å and 1.3 Å for patch 1 and 2, respectively (Fig. 4B, C). The RMSF of unbound RBD will provide a baseline for comparing the fluctuations with different ligand bound complexes.

### Analysis of structural stability and binding free energy for Delta complexes

To better investigate the correct binding mode and estimate the stability of the predicted complexes, we performed unconstrained 100 ns MD simulations of the most promising 20 candidates within Delta RBD, followed by free energy of binding calculations using MM-GBSA. First, we monitored the conformational stability of the protein through MD simulations calculating Ca-RMSDs (Fig. S1). As observed from the RMSD values, MD simulations in each of the 20 simulated systems reach the equilibrium after about 10 ns, and structural fluctuations are maintained in the range of 1.3–2.5 Å. Then, extent of conformational fluctuations of selected hit molecules within the RBD was also investigated. For 16 complexes, the ligand did not retain its initial conformation within the RBD, dissociating from the protein or binding to a different region of protein’s surface. The RMSD of the ligand inside the binding site, with respect to its docking pose coordinates (RMSD_lig_) for the 20 simulated systems, are also shown in Fig. S1. Compounds #5, #7, #8, #9, #11, #13, #14, #18, #19, #20 dissociated from the docking site and interacted with a different region of the RBD surface; #1, #3, #6, #10, #12, #16 moved away from the RBD. Four ligands (#2, #4, #15, #17, Figs. S1 and 5) maintained a binding orientation like the docking pose (RMSD_lig_ < 5.5 Å) and have not diffused away from initial binding site, namely ZINC000003824921 (fexofenadine, compound #2), ZINC000035644633 (3'-hydroxy repaglinide, compound #4), ZINC000030731084 (RPR121056-d3, compound #15) and ZINC000150339331 (hydroxy itraconazole, compound #17).

**Figure 5:**
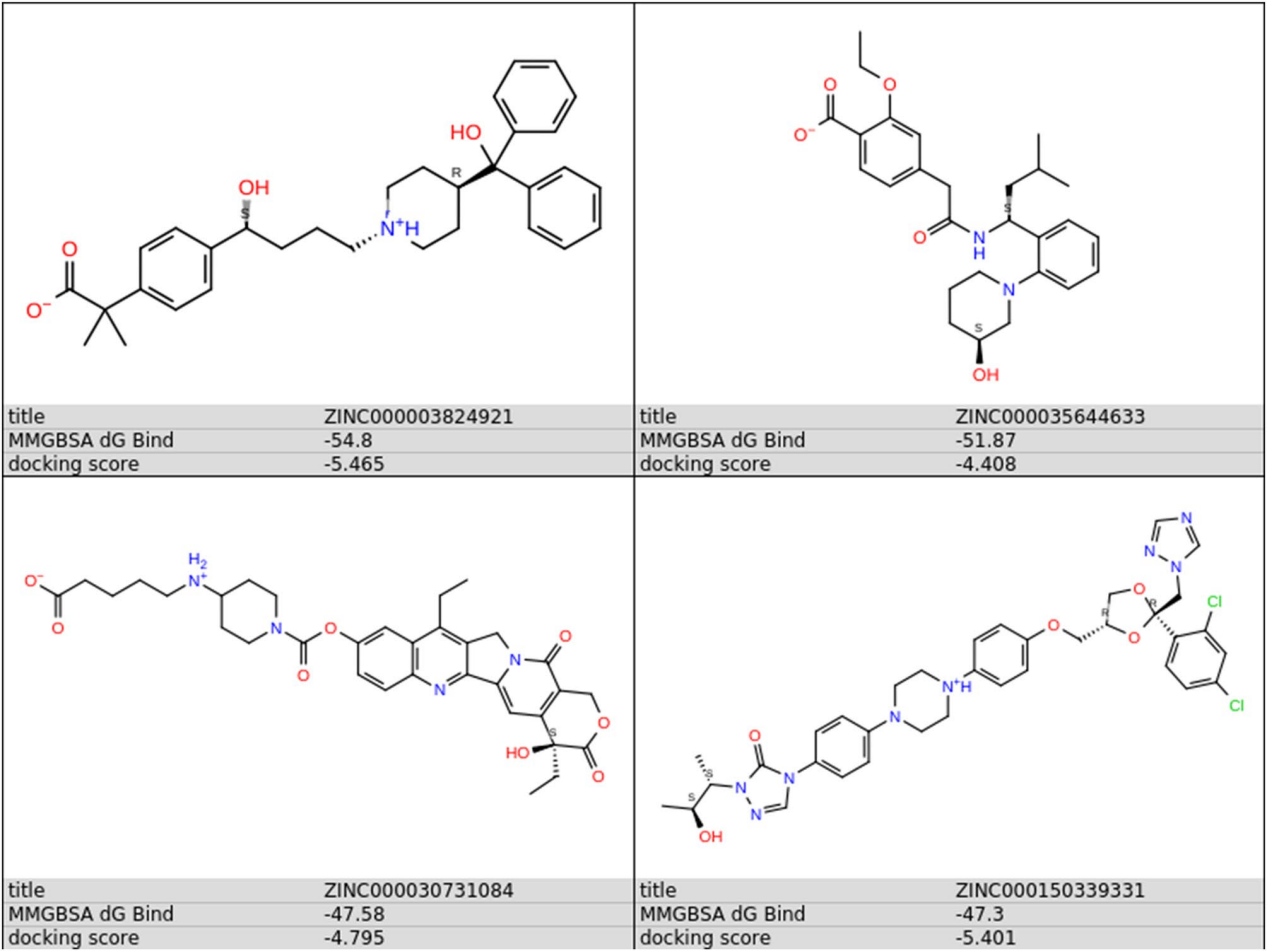
The chemical structures of the compounds remaining bound to the predicted binding pocket in the MD simulation.

**Figure 6:**
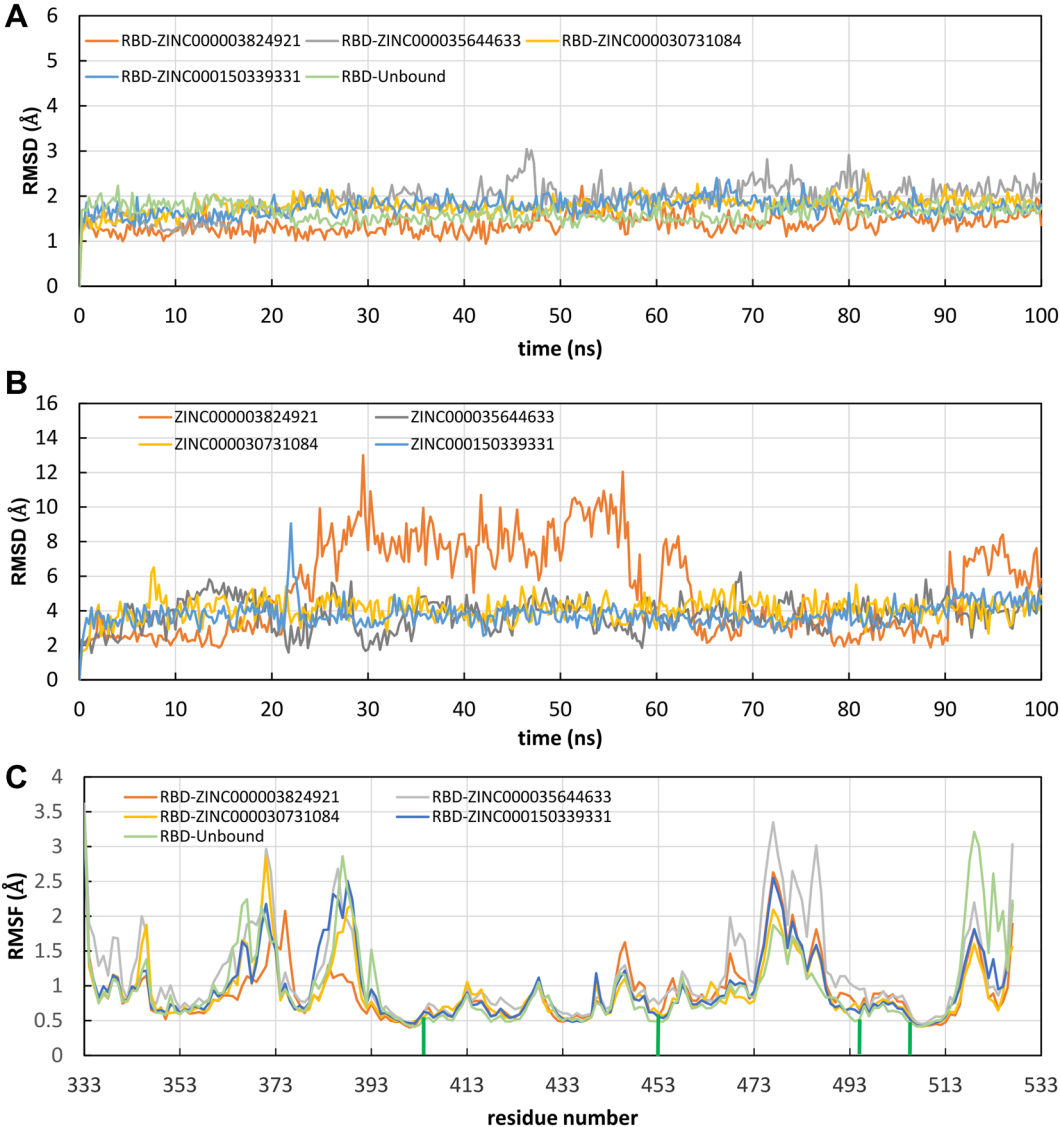
Evolution of structural properties over time. (**A**) Stability of the predicted complexes represented as the time evolution of the Cα-RMSD. (**B**) Variation of ligand RMSD with time. (**C**) Residue-based Cα-RMSF calculated over the entire trajectory. The green bars indicate the main contacts involved in molecular recognition.

Compounds #4, #15 and #17 are very stable within the predicted binding site for the total simulation time. Compound #2 remained bound to the binding pocket, but while its hydroxy(diphenyl)methyl moiety (Fig. 5) forms stable hydrophobic and hydrogen bonds interactions with Tyr505 and Gln493, respectively, all over the simulation time, the hydrogen bonding interactions of the methylpropionyl group of #2 with Arg452 or Ser494 were broken between 20 and 65 ns (giving rise to the increase of the RMSD_lig_) and reformed between 65 and 90 ns (Fig. S1). Superimposition of RMSD and RMSF plots for the stable complexes is shown in Fig. 6. The Cα-RMSD of unbound RBD is also shown for comparison indicating that the protein’s structure did not change during the simulation time (Fig. 6A). The variation of RMSD_lig_ highlighted the considerable stability of the ligand position inside the binding pocket, and the major flexibility of fexofenadine (ZINC000003824921) (Fig. 6B).

In addition, the Cα-RMSF was also calculated to monitor structural fluctuations and get further insight about the binding mode analysis of selected ligands. It is important to point out that also the RMSF plots of the complexes were highly similar to that of RBD (Fig. 6C). This was especially remarkable for those residues critically involved in molecular recognition, where least fluctuations were observed (green bars in Fig. 6C). Analysis of trajectories indicated that the selected drugs remained close to their initial positions obtained from docking analysis, confirming the formation of stable complexes.

**Figure 7:**
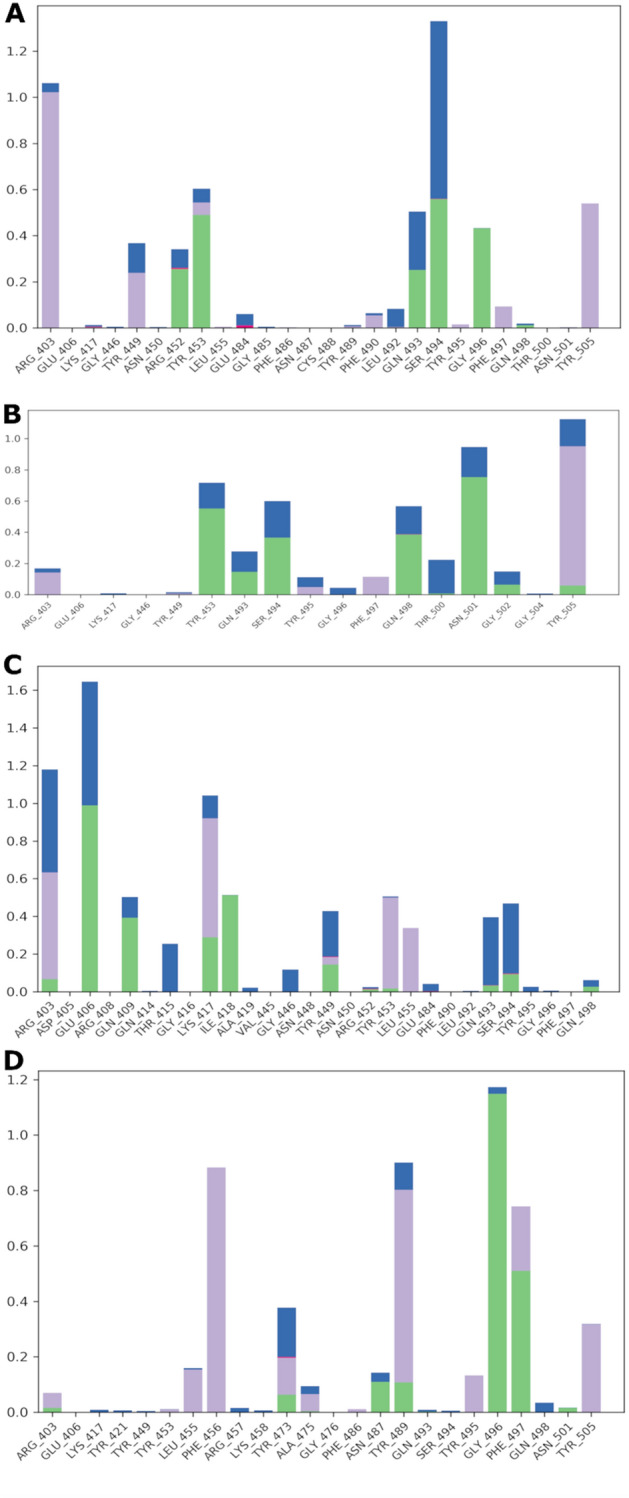
Spike RBD interactions with compounds #2 (**A**), #4 (**B**), #15 (**C**), #17 (**D**) monitored throughout the simulation trajectories. The interactions are clustered by type and shown in bar diagram including H-bonds (green), hydrophobic (light purple), ionic (pink) and water bridges (blue).

Simulation interactions diagrams during the entire MD run provided insights into the interaction pattern of the four ligands with Delta RBD. Figure 7 shows those interactions occurring during the MD simulation. Ligand #2, #4 and #15 exhibited hydrogen bonding and water bridges with Spike RBD through Gln493 and Ser494 and hydrogen bonding interactions with Tyr453. Hydrophobic interactions of #2, #4, #17 ligands with Tyr505 were also observed. Important hydrophobic interactions were observed for #17 with Phe456, Tyr489 and Tyr505. As it can be seen from Table 3 #17 exerted the most favored MM-GBSA energy (− 74.8 kcal/mol), with almost 18 kcal/mol, 24 kcal/mol and 27 kcal/mol difference comparing to #2 (− 56.4 ± 12.0), #4 (− 50.8 ± 16.7) and #15 (− 46.9 ± 4.6), respectively. The favored binding of hydroxy itraconazole can mainly be attributed to the lipophilic term of the binding energy (ΔG_Lipo_) and to the non-polar (ΔG_SolvSA_) contribution to the solvation free energy (Table 3).

**Table 3:** Prime MM-GBSA energies (kcal/mol) for ligands binding at the Delta Spike RBD.

ligand	^a^ΔG_bind_	^b^ΔG_Coulom_	^c^ΔG_Covalent_	^d^ΔGH_bondd_	^e^ΔG_Lipo_	^f^ΔG_Packing_	^g^ΔG_SolvGB_	^h^ΔG_SolvSA_	^i^ΔG_vdW_
#2	− 56.4 ± 12.0	− 9.7	2.6	− 1.5	− 20.0	− 3.9	12.2	− 36.1	5.3
#4	− 50.8 ± 16.7	− 46.8	2.7	− 1.8	− 16.6	− 3.1	47.6	− 32.9	9.2
#15	− 46.9 ± 4.6	− 2.1	2.3	− 2.2	− 13.0	− 1.4	12.2	− 42.7	5.3
#17	− 74.8 ± 7.2	− 10.3	0.2	− 1.3	− 23.1	− 8.0	16.2	− 48.5	11.1

^a^Total free energy of binding, in kcal/mol as calculated by the MMGBSA method, averaged over the time of simulation.

^b^Electrostatic Coulomb term of the binding energy.

^c^Covalent term of the binding energy.

^d^Hydrogen bond contribution to the binding energy.

^e^Lipophilic contribution to the binding energy.

^f^π–π packing correction.

^g^Generalized Born term of the solvation energy.

^H^non-polar contribution (SA, Surface Area) to the solvation energy.

^i^van der Waals term of the binding energy.

### Benchmark test

To investigate the performance of the computational protocol we conducted a benchmark study on a set of 11 inhibitors of the Delta Spike RBD-ACE2 interaction with IC50 values determined experimentally. First the set was screened against the CNN assisted QSAR model which classified all the benchmark ligands as PPI modulators. Molecular docking of the benchmark compounds was then performed to set a benchmark cutoff score for identifying novel inhibitors of the Spike RBD-ACE2 interaction. Prime MM-GBSA energies for benchmark ligands at the Spike RBD site are reported in Table 4. The predicted binding energies for the experimentally determined inhibitors using the MM-GBSA algorithm ranges between − 53.78 kcal/mol and − 32.92 kcal/mol. The free binding energy of the best four compounds against Delta (fexofenadine, 3'-hydroxy repaglinide, RPR121056-d3, and hydroxy itraconazole, binding energy values: − 54.80, − 51.87, − 47.58 and − 47.30 kcal/mol, respectively, Table 2) in comparison with the benchmark ligands suggest that our screening approach can achieve efficient inhibitor identification. Of note, more favorable binding energy was shown by fexofenadine when compared to the whole set of benchmark ligands.

**Table 4:** Prime MM-GBSA energies (kcal/mol) for benchmark ligands.

ligand	Predictive ability of the CNN-based QSAR model	ΔGbind
Pixatimod^27^	True	− 53.78
AB-00011778^28^	True	− 47.45
CgRd^29^	True	− 43.86
DRI-C23041^29^	True	− 41.17
DRI-C41041^29^	True	− 40.44
DRI-C24041^29^	True	− 38.08
TBrC^30^	True	− 36.39
DV1^29^	True	− 35.09
Compound5^31^	True	− 34.96
DRI-C2105041^29^	True	− 34.41
EvBl^29^	True	− 32.92

Molecular dynamics simulations of the best two benchmark ligands (Pixatimod and AB-00011778, Table 4) bound at the RBD binding site were also performed. Plots showing the stability of the ligands within the predicted site and comparison with the selected four drugs (fexofenadine, 3'-hydroxy repaglinide, RPR121056-d3, and hydroxy itraconazole) are presented in Supplementary Information (Figs. S2–S4). In all these systems the distance between the center of mass of the ligands and the center of mass of the Delta Spike RBD site remains constant over the simulation time (Supplementary Information, Figs S2 and S4).

### Activity prediction on Omicron

**Figure 8:**
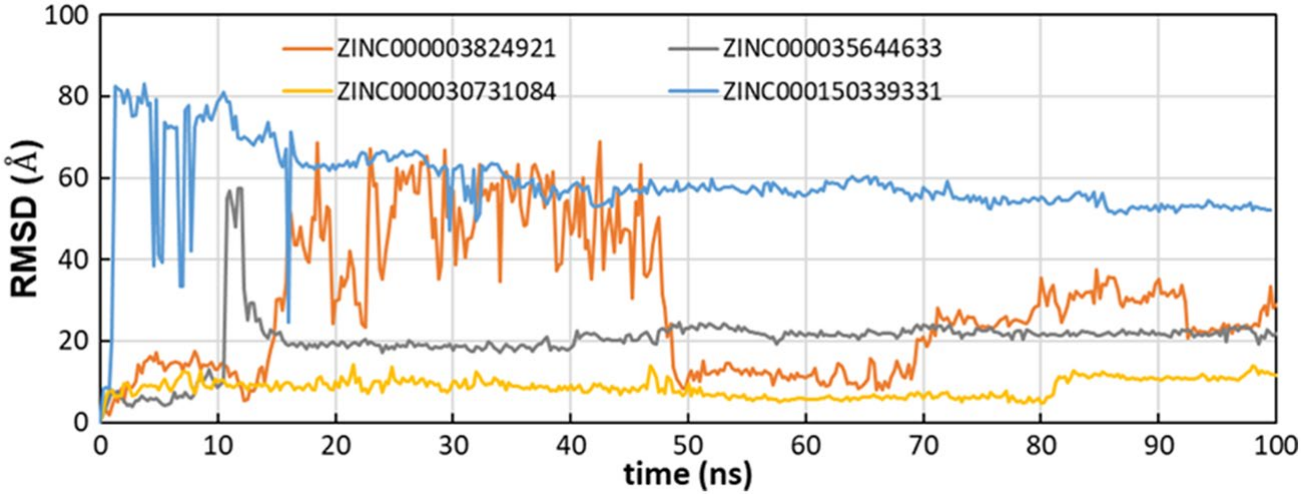
Time evolution of RMSD_lig_ in the Omicron complexes.

**Figure 9:**
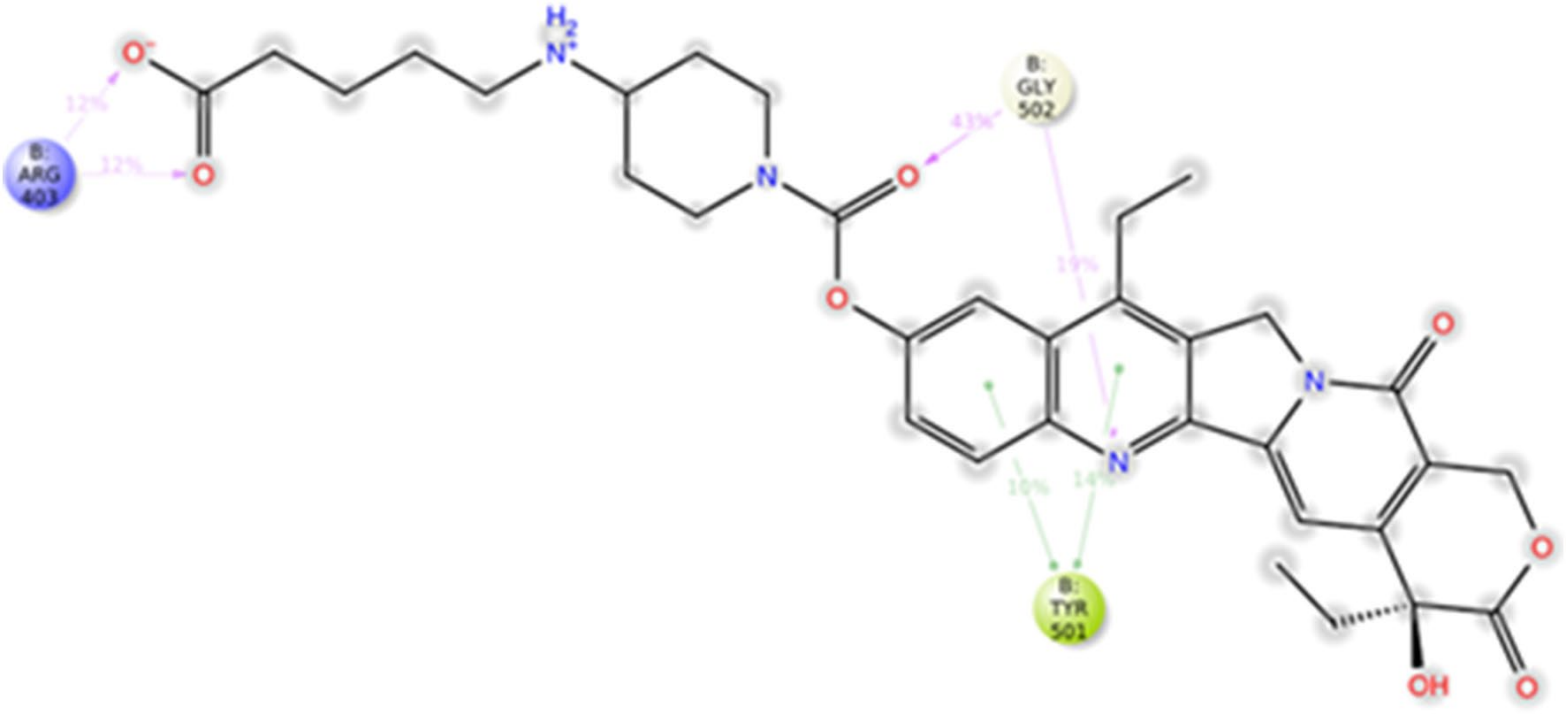
A schematic representation of the intermolecular interactions between #15 and Omicron Spike RBD.

The best four compounds against Delta have been docked against Omicron RBD in the predicted binding pocket and the resulting complexes submitted to MD simulations. MM-GBSA binding energies from docking were − 63.84 kcal/mol, − 51.70 kcal/mol, − 50.91 kcal/mol and − 48.26 kcal/mol for ZINC000150339331 (hydroxy itraconazole, compound #17), ZINC000003824921 (fexofenadine, compound #2), ZINC000030731084 (RPR121056-d3, compound #15) and ZINC000035644633 (3'-hydroxy repaglinide, compound #4), respectively. MD trajectories showed that compounds #2, #4 and #17 moved away from the initial position during the MD simulation (average RMSD_lig_ were 28.7 ± 16.9 Å, 20.2 ± 6.5 Å and 58.6 ± 9.7 Å, respectively). Compound #15 showed an average value of 9.1 ± 1.6 Å in the first 50 ns, 6.3 ± 0.7 Å between 50 and 80 ns and 10.8 ± 1.7 Å from 80 ns to the end of simulation (Fig. 8). Compound #15 was stable at the Omicron RBD-ACE2 interface, by means of hydrogen bonds with Arg403 and Gly502 and hydrophobic interactions with Tyr501 (Fig. 9). These data were corroborated by binding free energies values of the four selected compounds to the Omicron RBD calculated by the MM-GBSA method using the snaphots extracted from the MD trajectories. Table 5 shows that the first ranked compound was compound #15 and that hydrophobic interactions provide a favorable factor for association with Omicron RBD.

**Table 5:** Prime MM-GBSA energies for ligands binding at the Omicron Spike RBD.

ligand	^a^ΔGbind	^b^ΔG_Coulom_	^c^ΔG_Covalent_	^d^ΔG_Hbondd_	^e^ΔG_Lipo_	^f^ΔG_Packing_	^g^ΔG_SolvGB_	^h^ΔG_vdW_
#2	− 9.1 ± 13.3	− 8.4	0.7	− 0.5	− 3.2	− 0.5	11.9	− 9.1
#4	− 23.3 ± 16.5	− 26.7	0.6	− 0.7	− 8.5	− 0.6	32.2	− 19.6
#15	− 35.2 ± 6.6	− 7.2	3.3	− 1.8	− 10.8	− 2.1	14.3	− 30.8
#17	− 23.0 ± 26.5	− 10.3	0.3	− 0.3	− 8.0	− 1.8	16.8	− 19.7

^a^Total free energy of binding, in kcal/mol as calculated by the MM-GBSA method, averaged over the time of simulation.

^b^Electrostatic Coulomb term of the binding energy.

^c^Covalent term of the binding energy.

^d^Hydrogen bond contribution to the binding energy.

^e^Lipophilic contribution to the binding energy.

^f^π–π packing correction.

^g^Generalized Born term of the solvation energy.

^h^van der Waals term of the binding energy.

## Discussion

The management of SARS-CoV-2 post-pandemic needs general health strategies also accounting for the possibility that this infection will become endemic^32,33^. In this context the vast research effort done so far, that must be celebrated for the great human achievements, should now give way to a new research approach, by means of fast pipelines for drug discovery and repositioning and social policies. The continuous variation of the Spike protein in fact causes concern of risk in terms of transmissibility and/or pathogenicity, also in population with high prevalence of vaccinated individuals. In this regards an unmet need is represented by the availability of fast methodological approach able to promptly deepen the features of the putative novel variants and hopefully to predict the efficacy of molecules/drugs to be repurposed.

The most successful drugs currently approved for clinical use, have been predicted and deepened in their mechanisms of action also through computational methodologies, such as in silico screenings^34,35^, immunoinformatic and structure-based drug design^36–38^. Several strategies have been approached to then identify and confirm computationally predicted molecules or to repurpose FDA-approved drugs to counteract the SARS-CoV-2 pandemic. Since the emergence of this infectious disease, a variety of in vitro methodology has been performed, mainly based on infected Vero^39^, HEK^37^, Calu-3, Huh7 cells^40^ and hPSC lung organoids^41^, among the wide amount of other cell types. Very few in vitro tested molecules progressed to in vivo models and clinical studies and only a very small amount of them were successful at different phases of clinical trials.

Through these different methodologies, it has been possible to dispose of antivirals such as: remdesivir approved by FDA after multiple and independent clinical trials (NCT04280705, NCT04292730, NCT04292899, https://www.accessdata.fda.gov/drugsatfda_docs/label/2020/214787Orig1s000lbl.pdf); molnupiravir (NCT04746183, NCT04939428, NCT04405739, https://www.fda.gov/media/155053/download), nirmatrelvir and ritonavir (NCT04960202, https://www.fda.gov/media/155049/download). These antivirals were authorized by FDA for emergency use in the treatment of mild-to-moderate infected adults or children (older than 12 years), with high risk for progression to severe symptoms, including hospitalization or death. FDA also approved several kinase inhibitors, mainly for emergency use and in combination with other validated treatment(s), such as baricitinib, entrectinib and imatinib (NCT04422678^42^) and drug transporters such as equilibrative nucleoside transporters (ENT) 1 and 2^43^.

Computational methods allowed not only to deepen the interacting properties of SARS-CoV-2 Spike with the ACE2, but also to evaluate a variety of natural and synthetic compounds as potential small-molecule specific inhibitors of Spike protein-ACE2 binding. Starting from these results and aware to transitioning to an era of endemicity with SARS-CoV-2 research, in this work we purpose a computational strategy for predicting if the therapeutic agents identified for past variants may continue to be useful against new ones. To the best of our knowledge few studies reported the use of molecular docking for repurposing already approved drugs as inhibitors of the interaction between Spike-RBD and the ACE2 receptor. For example, in 2020 a high-throughput virtual screening campaign of the University of Tennessee identified nitrofurantoin and isoniazid as potential SARS-CoV-2 treatment agents^44^. Targeting Spike-RBD and combining molecular docking with dynamics simulations few good anti-COVID-19 candidates (gonadorelin^17^, fondaparinux^17^, atorvastatin^17^ and the antiviral atovaquone^45^ and praziquantel^45^) were identified. In silico screening against ACE2 identified cefpiramide as a potential inhibitor of Spike protein-ACE2 binding^46^. The advantage of our approach is the use of a subset of the library of approved drugs which includes compounds with high probability to modulate the PPI interface and inhibit the infection in the host cells.

From the site identification analysis, one pocket was identified at the Delta- and Omicron-ACE2 interface embedding hotspot residues. This pocket is shallow, and, from the MD simulations, we found that most predicted ligands were unable to form stable interactions with cavity-lining residues, this result confirming the importance of corroborating docking data by dynamic analysis. Here we used different approaches to select the compounds including rescoring, MM-GBSA, and a machine learning model to try to distinguish molecules able to interfere with the Spike RBD/ACE2 interaction. The computational pipeline gave us four top-hits, which exhibited good binding affinity towards Delta RBD. The compound with the most interesting features is fexofenadine, an antihistaminic drug already in use for the treatment of seasonal allergies (hay fever), skin itching and chronic idiopathic urticaria in adults and children^47^. Remarkably, antihistaminic drugs were found to exhibit direct antiviral activity against SARS-CoV-2 in vitro^48^. Fexofenadine, together with other histamine agonists, was also proposed as a potential COVID-19 drug by molecular docking studies against main protease in 2020 by Singh and co-workers^49^. Moreover, it has been demonstrated that patients hospitalized with COVID-19 have had benefits after the administration of a single or combined antihistaminic drugs^50,51^, correlating with increased survival and halted COVID-19 symptom progression, especially in the ones with critical conditions^52^. It has been suggested that histamine agonists could be able to down-regulate the excessive cytokine release, although the potential mechanism(s) of action about their impact on COVID-19 symptoms is(are) still unclear. Similarly, it is debated their usage in persons experiencing long-Covid symptoms (PASC, Post-Acute sequelae of SARS-CoV-2), where anecdotal descriptions have been reported^53^. Our data on the one hand corroborate these clinical reports, suggesting and giving new insights into the putative mechanism(s) of action of antihistaminic drugs on SARS-CoV-2, and, on the other hand, highlight the potential of this methodology for a rapid screening of variant-specific drugs.

Other compounds showed a good inhibitory ability against Spike-ACE2 complex both for Delta and Omicron variants: repaglinide, a hypoglycemic drug, approved for the treatment (in combination with rosiglitazone or pioglitazone) of type 2 diabetes^54^; RPR121056-d3, a metabolite of Irinotecan (CPT-11), already approved as antineoplastic agent^55^; itraconazole, already approved for the treatment of certain fungal infections (candidiasis and histoplasmosis)^56^.

Fexofenadine has been purposed as a potential target drug for COVID-19 treatment^49,57^. This substance has antihistamine properties and is indicated for the symptoms of allergies, through blocking of H1 receptors. In SARS-CoV-2 we agree with previous works that hypothesized that fexofenadine could impact indirectly on the cytokine storm by inhibiting histamine and, consequently, interleukin-6 (IL-6) production, directly on the viral replication, interacting on the protease enzyme MPro^49,58^. A similar direct mechanism of action has been proposed for repaglinide, an antidiabetic drug, that in analogy to other antiviral drugs (e.g., nelfinavir) has been candidate as a potential inhibitor of the MPro. Further experiments would be needed to determine the mechanism(s) of action of these two drugs. While RPR121056-d3 has not yet been reported as a potential treatment of COVID-19, itraconazole showed an in vitro activity against SARS-CoV-2, possibly by inhibiting oxysterol-binding protein that interferes with intracellular lipid transfer^59^ and by favoring the cholesterol accumulation in the endosomal membrane preventing the viral transfer into the target cells^60^.

Interestingly all these drugs, although with different mechanism of actions and with different composition, are already used to modulate immunopathologic processes, including fexofenadine, which is recommended for immune-mediated respiratory diseases, more specifically in upper airway diseases and allergic rhinitis^61^. Our results from prediction and in silico studies have thus succeeded in selecting drugs that, with distinct suggested or proven mechanisms of action, appear to be able to interfere with the virus' ability to infect and damage host cells. To date one of the unmet needs in counteracting the spread of SARS-CoV-2 and the generation of new variants is represented by the control of novel or recurrent infections, both for adults and children and for fragile and elderly patients^62–65^. In this regard, among the selected drugs, fexofenadine in a ready-to-use spray formulation, could be a valid option, as suggested also for other drugs^66^. These drugs, identified via computational approaches can be assessed experimentally and provide a basis to develop novel treatment options against COVID-19 variants.

## Materials and methods

### Proteins preparation

Crystallographic^26^ and cryo-EM^26^ structures of SARS-CoV-2 Delta and Omicron RBD, bound to ACE2, were downloaded from the protein data bank (PDB) (PDB codes: 7WBQ and 7WBL, respectively) and used as targets for virtual screening. RBDs were extracted from the PDB structures of ACE2-RBD complexes and prepared using the “Protein Preparation Wizard” tool of the Schrödinger suite (Schrödinger Release 2021-4: Protein Preparation Wizard; Epik, Schrödinger, LLC, New York, NY, 2021). The protocol included, following water molecules and cofactors removal, correcting mislabeled elements, adding hydrogen atoms, assigning bond orders, hydrogen bond optimization, and restrained energy minimization using OPLS4 force field^67^. The prepared proteins were then considered for grid generation using the “Receptor Grid Generation” panel of the Glide module of the Schrödinger suite (Schrödinger Release 2021-4: Glide, Schrödinger, LLC, New York, NY, 2021). The center of each grid (size 15 × 15 × 10 Å) was arranged at the centroid of the predicted druggable site using SiteMap^20,68^.

### Identification of druggable pockets

SiteMap was used to explore possible druggable pockets on Delta and Omicron RBD surfaces^69,70^. The program uses OPLS4 force field to estimate the interaction energies of probes placed at all points along a three-dimensional grid that encompasses the entire RBD with a minimum of 15 site points per site. SiteMap ranks identified sites using two druggability assessment scores: SiteScore and Dscore, which characterize the binding site in terms of size, exposure to solvent, hydrophobicity and hydrophilicity, degree of hydrogen bond donation and acceptance. The highest ranking regions were further selected for embedding hotspot residues at RBD-ACE2 surface^20^. Evaluation of the SiteMap results helped to locate the binding sites for docking calculations.

### Dataset and library preparation

The ZINC database is a free online database comprising more than 230 million purchasable compounds in ready-to-dock, 3D formats^71^ and is commonly used for virtual screening against enzymes and other targets^72,73^. The subset “World” of ZINC15, consisting of a total of 4388 small molecules, was downloaded. “World” includes drugs approved in major world jurisdictions, belonging to different collections: DrugBank^74^, HMDB^75^, Microsource International Drug Collection (http://www.msdiscovery.com/intdrug.html) and Therapeutic Targets Database^76^. The library was prepared by Schrödinger’s LigPrep tool of Maestro (Schrödinger Release 2021-3: LigPrep, Schrödinger, LLC, New York, NY, 2021), to apply the OPLS4 force field, to optimize the structures and to add hydrogen atoms. Epik, implemented within, was used to assign likely protonation states at pH 7 ± 2 and tautomers to each molecule. Prepared molecules, in SMILES (Simplified Molecular Entry Line Entry System) format, were screened against our published convolutional neural network (CNN) assisted QSAR model which was shown to identify potential PPI modulators among a virtual library of compounds^20,77^.

### Structure-based virtual screening

Virtual screening was performed using the Glide program (Schrödinger Release 2021-4: Glide, Schrödinger, LLC, New York, NY, 2021). A ligand-flexible docking was performed using the prepared library of compounds and the Virtual Screening Workflow (VSW) protocol of Glide. Molecular docking was performed at two different levels of precision, standard (SP) and the more accurate extra precision (XP) modes using default parameters. All docked compounds were rescored based on binding energy using the Prime/MM-GBSA (Schrödinger, LLC) method. The best 20 molecules for Delta system were selected and considered for molecular dynamics simulations of predicted complexes. A set of benchmark compounds was selected and treated in the same manner as described by the protocols for the library of approved drugs. The benchmark set includes 11 reported inhibitors of the Delta Spike RBD-ACE2 interaction with IC50 values in the micromolar to nanomolar range.

### Molecular dynamics calculations

All-atom molecular dynamics simulations were performed using the Desmond-6.8 module of Schrödinger software package (Schrödinger Release 2021-4: Desmond Molecular Dynamics System, D. E. Shaw Research, New York, NY, 2021) as implemented in Maestro. All docked complexes were placed in a cubic water box at a buffer distance of 10 Å and solvated with SPC water models. A 0.15 M NaCl salt concentration was added and additional Na^+^/Cl^−^ ions were added to neutralize the systems. The particle-mesh Ewald method was used to calculate the long-range electrostatic interactions. A cut-off radius of 9.0 Å was applied for short-range van der Waals and Coulomb interactions. Each solvated system was minimized and equilibrated using the default protocol of Desmond in Maestro which includes 2 NVT and 2 NPT restrained short simulations. All equilibrated systems were then subjected to a MD run with periodic boundary conditions in the NPT ensemble using OPLS4 force field^78^ for 100 ns. The temperature of 300 K and the pressure of 1 atm of the systems were maintained by the Nosè–Hoover chain thermostat and Martyna–Tobiase–Klein barostat methods, respectively. The binding energy between the Spike RBDs and the docked ligands was calculated over the 100 ns period with thermal_mmgbsa.py python script provided by Schrödinger which takes a Desmond trajectory file, splits it into individual snapshots, runs the Prime-MMGBSA calculations on each frame, and yields the average calculated binding energy. Best compounds for Delta RBD were also analyzed by molecular dynamics simulations on Omicron RBD. 100 ns MD simulations of the best benchmark ligands bound at the RBD site and 200 ns MD simulations of Delta RBD/ACE2 complex were also performed using the same conditions. Details of the starting systems for MD simulations are reported in Supplementary Materials (Table TS1).

## Conclusions

The possibility to identify and reposition drugs via computational analyses can address the current challenges related to the succession of waves of the infection of SARS-CoV-2 variants worldwide. This strategy allowed the prediction of selective compounds consistent with already suggested and prescribed drugs and is confirmed as a useful tool in support to clinical therapeutic approaches.

## Supplementary Information

Supplementary Information.

## Data Availability

Docking results and simulation trajectories datasets are freely accessible at zenodo.org as https://doi.org/10.5281/zenodo.6868548.
