# Publisher Correction: Virtual screening and molecular dynamics simulations provide insight into repurposing drugs against SARS-CoV-2 variants Spike protein/ACE2 interface

**DOI:** 10.1038/s41598-023-29766-8

**Published:** 2023-02-15

**Authors:** Davide Pirolli, Benedetta Righino, Chiara Camponeschi, Francesco Ria, Gabriele Di Sante, Maria Cristina De Rosa

**Affiliations:** 1 Institute of Chemical Sciences and Technologies ‘‘Giulio Natta’’ (SCITEC)-CNR, 00168 Rome, Italy; 2 Department of Translational Medicine and Surgery, Section of General Pathology, Università Cattolica del Sacro Cuore, 00168 Rome, Italy; 3 Fondazione Policlinico Universitario A. Gemelli IRCCS, 00168 Rome, Italy; 4 Department of Medicine and Surgery, Section of Human, Clinic and Forensic Anatomy, University of Perugia, 06132 Perugia, Italy

Correction to: *Scientific reports* 10.1038/s41598-023-28716-8, published online 27 January 2023

The original version of this Article contained errors in Table 2, where data in the heading row was displaced. As a result, the values in the columns ΔG_bind_, ΔG_Coulomb_, ΔG_Covalent_, ΔG_Hbondd_, ΔG_Lipo_, ΔG_SolvGB_ and ΔG_vdW_ appeared under an incorrect column heading. Additionally, the column heading “ΔG_Coulomb_” was incorrectly stated as “ΔG_Coulom_”. The original Table 2 and accompanying legend appear below.

**Table 2:** Binding energy (kcal/mol) and individual energy terms of Delta RBD-ligand systems calculated by Prime MM-GBSA.

ligand	ΔG_bind_	ΔG_Coulom_	ΔG_Covalent_	ΔG_Hbondd_	ΔG_Lipo_	ΔG_SolvGB_	ΔG_vdW_	
#1	ZINC000000004319	− 59.54	0.12	1.23	− 2.94	− 13.79	− 15.71	− 26.22
#2	ZINC000003824921	− 54.8	− 37.26	4.57	− 3.47	− 18.48	35.68	− 31.99
#3	ZINC000067665085	− 54.76	− 18.6	1.33	− 2.31	− 17.91	28.05	− 42.13
#4	ZINC000035644633	− 51.87	− 57.74	3.44	− 2.32	− 17.69	61.48	− 34.59
#5	ZINC000085540202	− 51.64	6.63	5.41	− 1.82	− 15.39	3.93	− 47.05
#6	ZINC000000607910	− 51.46	− 21.8	− 0.94	− 2.45	− 13.77	19.15	− 25.85
#7	ZINC000000538509	− 50.78	4.91	2.05	− 1.6	− 17.78	− 3.98	− 30.29
#8	ZINC000053073961	− 50.18	41.13	3.07	− 1.16	− 17.26	− 33.41	− 36.14
#9	ZINC000003810860	− 50.05	− 26.04	4.45	− 1.71	− 19.57	27.55	− 29.34
#10	ZINC000022446634	− 49.42	28.75	5.74	− 1.13	− 17.62	− 29.46	− 32.19
#11	ZINC000003816292	− 48.55	42.49	9.34	− 2.06	− 9.41	− 50.72	− 35.51
#12	ZINC000001530886	− 48.14	31.35	2.7	− 2.59	− 17.89	− 23.82	− 28.71
#13	ZINC000006716957	− 47.99	− 13.17	9.96	− 1.89	− 18.8	21.94	− 38.49
#14	ZINC000077313075	− 47.94	− 63.1	8.9	− 2.92	− 19.73	72.88	− 39.91
#15	ZINC000030731084	− 47.58	− 23.59	5.78	− 4.24	− 13.51	31.38	− 41.64
#16	ZINC000000537928	− 47.34	22.02	3.74	− 0.54	− 26.3	− 11.93	− 29.88
#17	ZINC000150339331	− 47.3	11.6	7.07	− 1.84	− 14.58	− 9.06	− 33.73
#18	ZINC000000001003	− 47.24	− 15.85	2.57	− 1.03	− 15.88	13.98	− 25.87
#19	ZINC000000608266	− 47.12	61.92	5.59	− 0.65	− 22.26	− 55.54	− 32.12
#20	ZINC000001552042	− 47.11	− 77.88	2.11	− 1.33	− 16.25	86.71	− 34.63

The original Article has been corrected.

