# Supplementary material for: Virtual screening and molecular dynamics simulations provide insight into repurposing drugs against SARS-CoV-2 variants Spike protein/ACE2 interface
Source: Sci Rep. 2023 Jan 27;13:1494. doi: 10.1038/s41598-023-28716-8 (PMC9880937; doi:10.1038/s41598-023-28716-8)

**Supplementary Information**

**Virtual screening and molecular dynamics simulations provide insight into repurposing drugs against SARS-CoV-2 variants Spike Protein/ACE2 interface**

Davide Pirolli^1,+^, Benedetta Righino^1,+^, Chiara Camponeschi^1^, Francesco Ria^2,3^, Gabriele Di Sante^4^, Maria Cristina De Rosa^1,^*

^1^Institute of Chemical Sciences and Technologies ‘‘Giulio Natta’’ (SCITEC)-CNR, 00168 Rome, Italy

^2^Department of Translational Medicine and Surgery, Section of General Pathology, Università Cattolica del Sacro Cuore, 00168 Rome, Italy

^3^Fondazione Policlinico Universitario A. Gemelli IRCCS, 00168 Rome, Italy

^4^ Department of Medicine and Surgery, Section of Human, Clinic and Forensic Anatomy, University of Perugia, 06132 Perugia, Italy

^+^D.P. and B.R. have equally contributed to this work

*Corresponding author:

Maria Cristina De Rosa

Institute of Chemical Sciences and Technologies "Giulio Natta" (SCITEC)-CNR, 00168 Rome, Italy. email: mariacristina.derosa@cnr.it

**Table ST1**. Details of the starting systems for all MD simulations

| *system* | *number of atoms* | *water molecules* | *Cl^–^ ions* | *Na^+^ ions* | *Size (Å)* |
| --- | --- | --- | --- | --- | --- |
| Delta RBD/ACE2 | 89226 | 25477 | 71 | 71 | 82X124X88 |
| OmicronRBD/ACE2 | 93833 | 93833 | 75 | 75 | 82X130X88 |
| DeltaRBD/Ligand#1 | 38872 | 11911 | 33 | 33 | 73X73X73 |
| DeltaRBD/Ligand#2 | 35157 | 10666 | 30 | 30 | 70X70X70 |
| DeltaRBD/Ligand#3 | 35192 | 10682 | 30 | 30 | 71X71X71 |
| DeltaRBD/Ligand#4 | 35194 | 10681 | 30 | 30 | 71X71X71 |
| DeltaRBD/Ligand#5 | 35187 | 10669 | 30 | 30 | 71X71X71 |
| DeltaRBD/Ligand#6 | 35181 | 10685 | 30 | 30 | 71X71X71 |
| DeltaRBD/Ligand#7 | 35184 | 10681 | 30 | 30 | 71X71X71 |
| DeltaRBD/Ligand#8 | 35117 | 10654 | 30 | 30 | 71X71X71 |
| DeltaRBD/Ligand#9 | 35201 | 10689 | 30 | 30 | 71X71X71 |
| DeltaRBD/Ligand#10 | 35137 | 10664 | 30 | 30 | 71X71X71 |
| DeltaRBD/Ligand#11 | 38882 | 11914 | 33 | 33 | 73X73X73 |
| DeltaRBD/Ligand#12 | 35165 | 10671 | 30 | 30 | 71X71X71 |
| DeltaRBD/Ligand#13 | 35181 | 10679 | 30 | 30 | 71X71X71 |
| DeltaRBD/Ligand#14 | 35183 | 10678 | 30 | 30 | 71X71X71 |
| DeltaRBD/Ligand#15 | 35170 | 10668 | 30 | 30 | 71X71X71 |
| DeltaRBD/Ligand#16 | 35153 | 10667 | 30 | 30 | 71X71X71 |
| DeltaRBD/Ligand#17 | 35129 | 10652 | 30 | 30 | 71X71X71 |
| DeltaRBD/Ligand#18 | 35179 | 10686 | 30 | 30 | 71X71X71 |
| DeltaRBD/Ligand#19 | 35141 | 10661 | 30 | 30 | 71X71X71 |
| DeltaRBD/Ligand#20 | 35205 | 10691 | 30 | 30 | 71X71X71 |
| OmicronRBD/Ligand#2 | 38497 | 11768 | 33 | 33 | 73X73X73 |
| OmicronRBD/Ligand#4 | 38502 | 11768 | 33 | 33 | 73X73X73 |
| OmicronRBD/Ligand#15 | 38460 | 11745 | 33 | 33 | 73X73X73 |
| OmicronRBD/Ligand#17 | 38495 | 11759 | 33 | 33 | 73X73X73 |

**Figure S1.**Time evolution of the Cα-RMSD (blue) and RMSD_lig_ (red) of the top twenty ranked Delta complexes


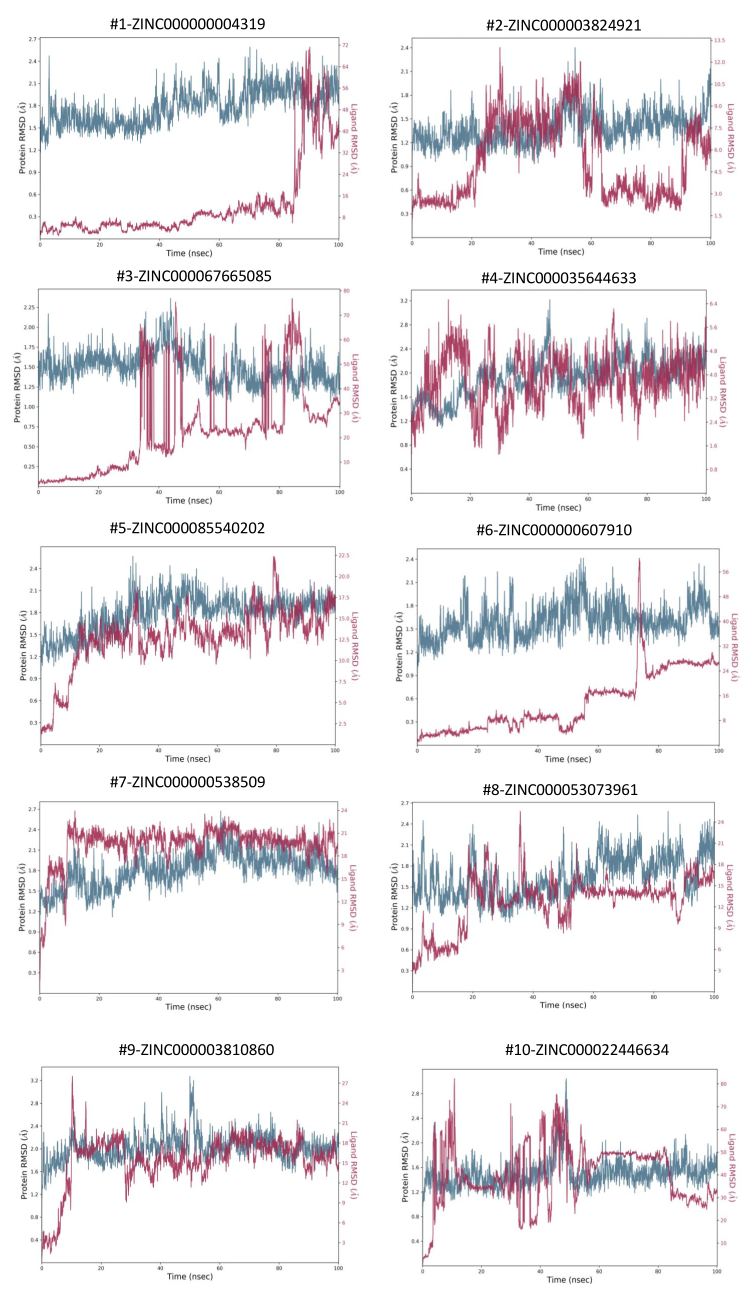


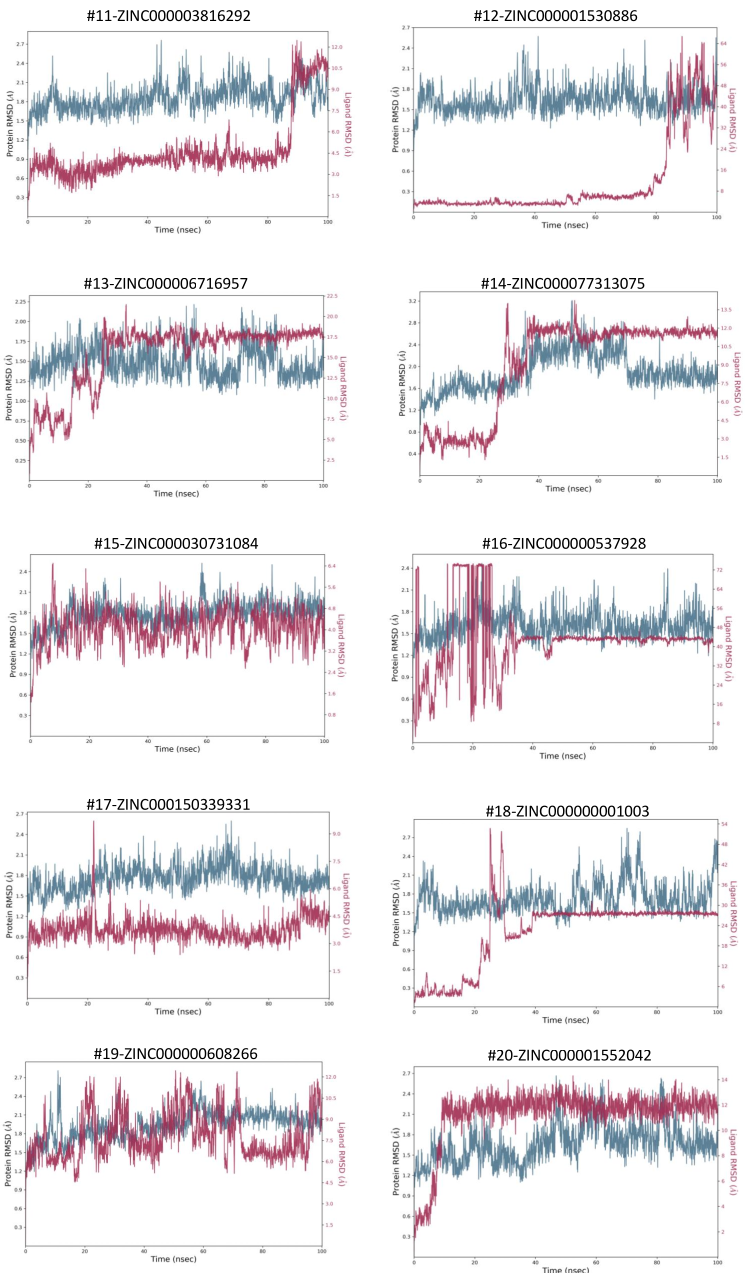


**Figure S2.** Time evolution of the distance between the center of mass of the Delta Spike RBD and the center of mass of compound #2, #4, #15 and #17, in blue, orange, grey and yellow, respectively.


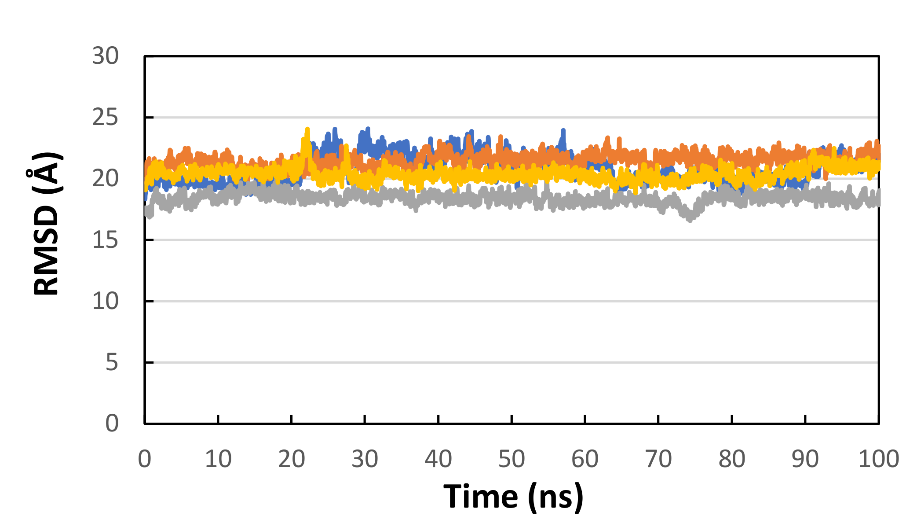


**Figure S3.** Time evolution of the RMSD_lig_ of Pixatimod (A) and AB-00011778 (B).


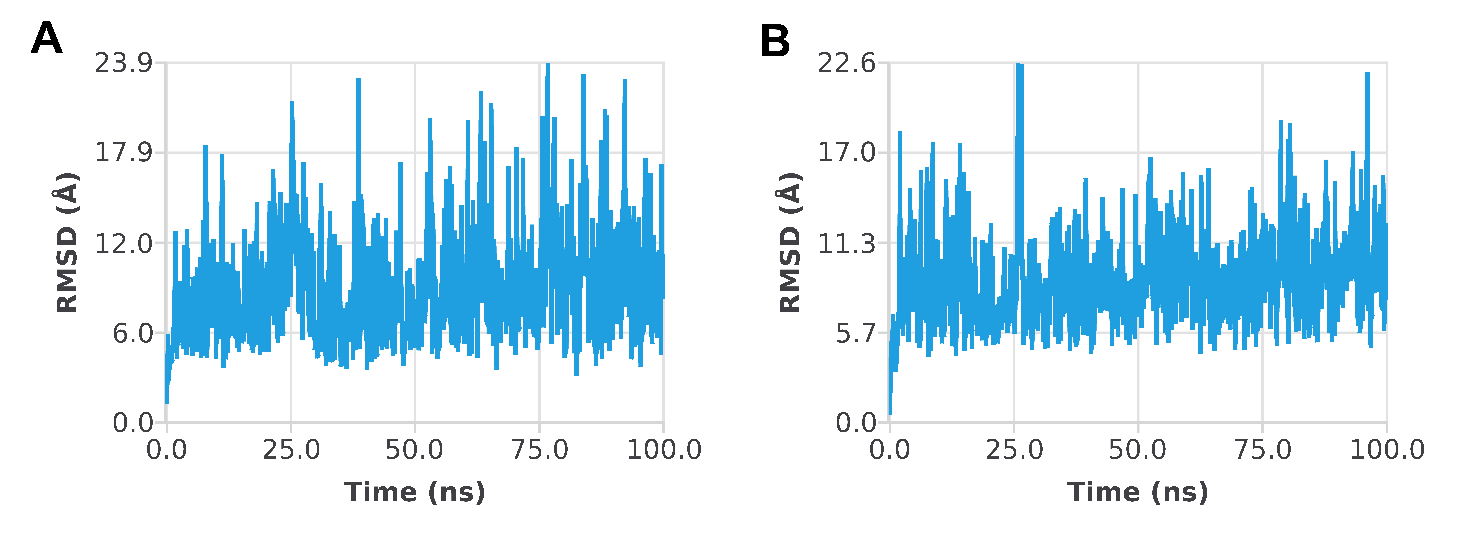


**Figure S4.** Time evolution of the distance between the center of mass of the Delta Spike RBD and the center of mass of Pixatimod and AB-00011778 in green and red, respectively.


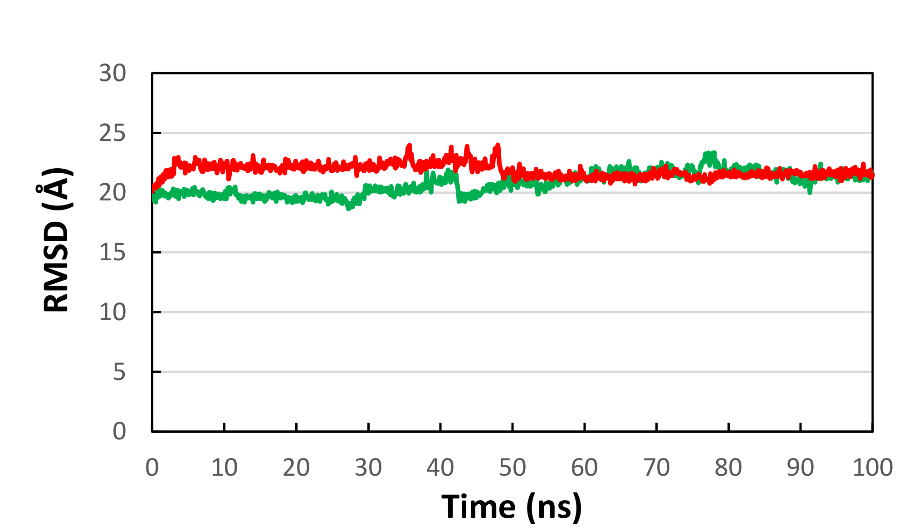

Supplement: Supplementary file 1 — Supplementary Information. [file 41598_2023_28716_MOESM1_ESM.docx]
